# Clinical outcomes and functional preservation of microwave ablation versus surgical resection for benign thyroid nodules: a propensity score-matched retrospective cohort study

**DOI:** 10.3389/fendo.2026.1822597

**Published:** 2026-08-13

**Authors:** Wu Xiao, Lianghua Luo, Bing Yao, Yong Yang, Wei Chen

**Affiliations:** 1 Department of Thyroid and Head-Neck Surgery, Jiangxi Provincial People’s Hospital, The First Affiliated Hospital of Nanchang Medical College, Nanchang, Jiangxi, China; 2 Department of General Surgery, Anfu County Hospital of Traditional Chinese Medicine, Ji’an, Jiangxi, China

**Keywords:** benign thyroid nodule, microwave ablation, propensity score matching, surgical resection, thyroid hormone dependence, volume reduction rate

## Abstract

**Background:**

Microwave ablation (MWA) is an organ-preserving minimally invasive option for symptomatic benign thyroid nodules. Comparative evidence on thyroid function preservation after MWA versus surgical resection (SR) remains limited, particularly when postoperative hormone replacement is separated by surgical extent.

**Methods:**

We retrospectively analyzed 113 consecutive adults treated with ultrasound-guided MWA or SR for symptomatic or cosmetically concerning cytologically benign thyroid nodules between January 2022 and October 2024. Patients with abnormal preoperative thyroid function, hyperfunctioning nodules, known autoimmune thyroiditis, prior thyroid surgery, indeterminate or malignant cytology, pregnancy, or concurrent thyroid malignancy were excluded. One-to-one propensity score matching was performed using eight pre-specified baseline covariates, yielding 48 matched pairs. The primary outcome was thyroid hormone dependence at 12 months, reported as overall dependence and, separately, as new-onset hypothyroidism after organ-preserving procedures. Secondary outcomes included operative duration, blood loss, hospital stay, 12-month symptom score, complications, recurrence, and volume reduction rate (VRR) after MWA.

**Results:**

After matching, baseline characteristics were well balanced. MWA was associated with shorter operative duration (median 18.0 vs. 63.5 minutes), lower blood loss (1.5 vs. 53.5 mL), and shorter hospital stay (1.0 vs. 5.0 days; all P < 0.001). In the MWA group, median VRR reached 86.2% at 12 months. Symptom scores improved substantially from baseline in both groups (both P < 0.001), with comparable low 12-month symptom burden. Overall thyroid hormone dependence occurred in 1 of 48 MWA patients (2.1%) and 25 of 48 SR patients (52.1%). In the SR group, this included planned replacement after total thyroidectomy (14/14, 100%) and new-onset hypothyroidism after lobectomy or subtotal thyroidectomy (11/34, 32.4%). In the sensitivity analysis excluding total thyroidectomy, new-onset hypothyroidism remained lower after MWA (2.1% vs. 32.4%; P < 0.001).

**Conclusions:**

In selected patients with small-to-moderate symptomatic benign thyroid nodules, MWA achieved substantial nodule shrinkage and low symptom burden at 12 months while reducing perioperative burden and postoperative hormone dependence. These findings support MWA as an organ-preserving alternative to surgery in appropriately selected patients, although randomized multicenter studies with longer follow-up are needed.

## Introduction

Benign thyroid nodules are common and usually require no intervention (1–3). Treatment is considered when nodules cause compressive symptoms, cosmetic concerns, or patient anxiety after malignancy has been excluded (4). Conventional surgical resection provides definitive anatomical removal, but it may require general anesthesia, hospitalization, a cervical incision, and, depending on the extent of resection, postoperative thyroid hormone replacement (5–8).

Image-guided thermal ablation has emerged as an organ-preserving alternative for selected benign thyroid nodules. Microwave ablation (MWA) produces coagulative necrosis within the target nodule while preserving uninvolved thyroid parenchyma (9, 10). The 2020 European Thyroid Association guideline and subsequent international guidance recognize thermal ablation as an option for symptomatic, cytologically benign nodules in appropriately selected patients and experienced centers (11–13).

However, most comparative studies of MWA and surgery remain retrospective, and the interpretation of postoperative thyroid function is complicated by heterogeneity in surgical extent. In particular, planned levothyroxine replacement after total thyroidectomy should not be interpreted in the same way as unexpected new-onset hypothyroidism after lobectomy, subtotal thyroidectomy, or MWA. Evidence that separates these clinically distinct outcomes is still limited.

This study compared MWA with surgical resection for symptomatic benign thyroid nodules using propensity score matching to reduce measured baseline imbalance. We focused on 12-month thyroid hormone dependence, while explicitly distinguishing planned replacement after total thyroidectomy from new-onset hypothyroidism after organ-preserving procedures. We also compared perioperative outcomes, symptom status, recurrence, complications, and nodule volume reduction after MWA.

## Methods

### Study design and participants

This retrospective cohort study was conducted at the Department of Thyroid and Head-Neck Surgery, Jiangxi Provincial People’s Hospital, The First Affiliated Hospital of Nanchang Medical College, Nanchang, China. Consecutive adults who underwent either ultrasound-guided percutaneous MWA or open SR for symptomatic benign thyroid nodules between January 2022 and October 2024 were identified from the institutional thyroid procedure registry. The data freeze date was December 31, 2025, ensuring that all included patients had completed 12-month follow-up. The study was approved by the Medical Ethics Committee of Jiangxi Provincial People’s Hospital (approval number: KK-2026-014; approved February 10, 2026), and the requirement for written informed consent was waived because of the retrospective design.

Inclusion criteria were: age 18 years or older; cytologically confirmed benign thyroid nodule on fine-needle aspiration biopsy (Bethesda category II); compressive symptoms, cosmetic concern, or both documented by the treating clinician; no prior thyroid surgery; American Society of Anesthesiologists physical status class I or II; and completion of 12-month clinical, ultrasound, and symptom-assessment follow-up. Exclusion criteria were malignant or indeterminate cytology (Bethesda III-VI), hyperfunctioning nodule on radionuclide scan, abnormal preoperative thyroid function, known autoimmune thyroiditis, coagulopathy or anticoagulation therapy not amenable to perioperative management, pregnancy, concurrent thyroid malignancy, or incomplete 12-month follow-up.

### Treatment procedures

MWA was performed under ultrasound guidance (Esaote MyLab Class C, 7.5–12 MHz linear transducer) by a single experienced operator. Local anesthesia with 2% lidocaine or monitored general anesthesia was selected according to patient preference, nodule location, and operator judgment. A coaxial 16-gauge microwave antenna (KY-2450B, Kangyou Medical, Nanjing, China) was inserted under real-time ultrasound guidance using a trans-isthmic approach. Ablation was performed at 40–60 W until the target nodule was covered by hyperechoic change on gray-scale ultrasound. Patients were observed after the procedure and discharged on the same or following day when clinically stable.

SR was performed under general anesthesia by board-certified endocrine surgeons through a standard Kocher incision. The extent of resection was individualized according to nodule distribution, contralateral nodular burden, patient preference, and surgeon judgment. In the matched cohort, lobectomy was performed in 25 patients, subtotal thyroidectomy in 9, and total thyroidectomy in 14. Total thyroidectomy was performed in patients with bilateral multinodular disease or substantial contralateral nodular burden; no patient underwent total thyroidectomy because of intraoperative conversion to malignancy. Intraoperative recurrent laryngeal nerve monitoring was used in all SR cases.

### Propensity score matching

To reduce measured selection bias, 1:1 propensity score matching was performed. Propensity scores were estimated using a multivariable logistic regression model with treatment group as the dependent variable and eight pre-specified baseline covariates as independent variables: age, sex, body mass index, maximum nodule diameter, preoperative nodule volume, baseline TSH level, hypertension, and diabetes mellitus. Maximum nodule diameter and preoperative nodule volume referred to the dominant (target) nodule rather than the total nodular burden in patients with multinodular disease. Nearest-neighbor matching without replacement was performed with a caliper width of 0.2 standard deviations of the logit of the propensity score using the MatchIt package in R. Covariate balance was assessed by standardized mean difference (SMD), with SMD <0.20 considered adequate balance and SMD <0.10 considered excellent balance. Because VRR is specific to ablation and surgery removes the nodule anatomically, VRR was reported only for the MWA group and was not compared statistically between groups. The primary matched cohort retained all surgical patients. To directly evaluate whether the functional advantage of MWA persisted against organ-preserving surgery alone, we additionally performed a separate 1:1 propensity score matching restricted to MWA versus organ-preserving procedures (lobectomy and subtotal thyroidectomy), using the same covariates, model, and caliper, with the smaller organ-preserving surgical group set as the index group for matching. Because the organ-preserving surgical pool was small (34 patients), this re-matched comparison was regarded as a secondary sensitivity analysis, and standardized mean differences were reported to document residual imbalance.

### Outcomes

The primary outcome was thyroid hormone dependence at 12 months, defined as the need for levothyroxine therapy at the 12-month follow-up visit. To avoid conflating planned replacement with unplanned thyroid failure, the outcome was reported in two layers. First, overall thyroid hormone dependence included planned replacement after total thyroidectomy and new-onset hypothyroidism after organ-preserving procedures. Second, new-onset hypothyroidism was analyzed after excluding total thyroidectomy patients from the SR group. New-onset hypothyroidism after MWA, lobectomy, or subtotal thyroidectomy was defined as TSH >4.5 mIU/L on at least two measurements obtained 4 or more weeks apart, with or without symptoms, leading to levothyroxine therapy.

Secondary outcomes included operative duration, intraoperative blood loss, length of hospital stay, symptom burden before treatment and at 12 months, residual or recurrent disease, and complications. Symptom burden was assessed using a 10-point patient-reported symptom scale, where 0 indicated no symptoms and 10 indicated the most severe symptoms, recorded both before treatment and at the 12-month visit. We reported the within-group change in symptom score from before treatment to 12 months for each treatment, together with the between-group comparison of pre-treatment and 12-month scores. Complications were classified using the Clavien-Dindo system and included hoarseness, hypocalcemia, hypothyroidism, hematoma, infection, and perioperative mortality. Residual or recurrent disease was defined as sonographic regrowth of the ablation zone or detection of an incompletely treated solid component at 12 months, confirmed by repeat fine-needle aspiration when clinically indicated.

### Follow-up and imaging protocol

Ultrasound follow-up examinations were performed at 3, 6, and 12 months after MWA by radiologists using a standardized institutional protocol. Nodule volume was calculated using the ellipsoid formula: V = π/6 × length × width × depth. VRR was calculated as [(preoperative volume - follow-up volume)/preoperative volume] x 100%. Thyroid function tests and serum calcium were obtained at 6 weeks, 6 months, and 12 months after treatment. All 96 matched patients completed 12-month follow-up.

### Statistical analysis

Statistical analyses were performed using R version 4.3.3 (R Foundation for Statistical Computing, Vienna, Austria). Continuous variables are presented as mean ± standard deviation or median with interquartile range according to distribution, and categorical variables are presented as frequency and percentage. Between-group comparisons used the independent-samples t test, Mann-Whitney U test, chi-square test, or Fisher exact test as appropriate. Within-group changes in symptom score from before treatment to 12 months were assessed using the Wilcoxon signed-rank test. P values equal to 1 were reported as >0.999. All tests were two-sided, and P <0.05 was considered statistically significant.

## Results

### Patient enrollment and baseline characteristics

**Figure 1:**
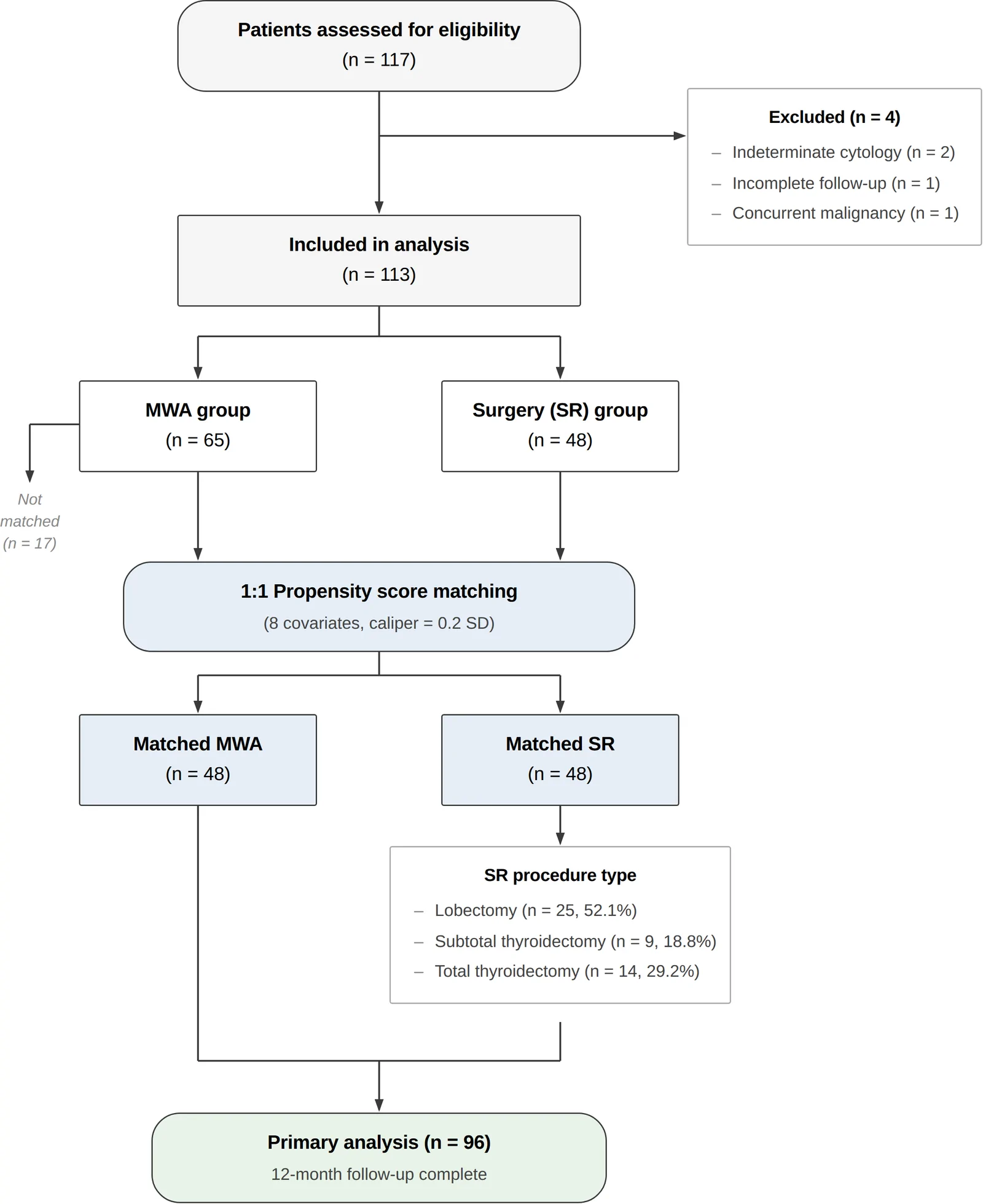
Patient enrollment and propensity score matching flowchart.

A total of 117 consecutive patients were assessed for eligibility. Four were excluded: two because of indeterminate cytology on central pathology re-review, one because of incomplete 12-month follow-up, and one because concurrent thyroid malignancy was identified. The unmatched cohort included 113 patients, 65 treated with MWA and 48 treated with SR. After propensity score matching, 48 matched pairs were included in the primary analysis (**Figure 1**).

Baseline characteristics before and after matching are shown in **Table 1**. After matching, all eight pre-specified covariates achieved adequate balance (SMD <0.20), and six achieved excellent balance (SMD <0.10). The matched MWA and SR groups had similar age, sex distribution, body mass index, maximum nodule diameter, preoperative volume, TSH level, hypertension, and diabetes.

**Table 1:** Baseline characteristics before and after propensity score matching.

Variable	MWA (n=65)	SR (n=48)	P value	SMD
Age (years)	46.4 ± 11.6	43.7 ± 10.1	0.190	0.248
Male sex, n (%)	13 (20.0)	10 (20.8)	>0.999	0.020
BMI (kg/m2)	23.7 ± 2.6	23.6 ± 3.6	0.852	0.036
Nodule size (mm)	25.2 ± 4.4	24.5 ± 5.7	0.469	0.141
Preoperative volume (mL)	3.1 ± 1.6	3.1 ± 2.5	0.884	0.029
TSH (mIU/L)	2.0 ± 1.1	1.9 ± 1.0	0.682	0.078
Hypertension, n (%)	10 (15.4)	6 (12.5)	0.788	0.083
Diabetes mellitus, n (%)	4 (6.2)	3 (6.2)	>0.999	0.004
Matched cohort				
Age (years)	42.3 ± 9.9	43.7 ± 10.1	0.475	0.146
Male sex, n (%)	11 (22.9)	10 (20.8)	>0.999	0.050
BMI (kg/m^2^)	23.6 ± 2.8	23.6 ± 3.6	0.962	0.010
Nodule size (mm)	24.4 ± 4.3	24.5 ± 5.7	0.954	0.012
Preoperative volume (mL)	2.9 ± 1.5	3.1 ± 2.5	0.626	0.100
TSH (mIU/L)	1.9 ± 1.0	1.9 ± 1.0	0.770	0.060
Hypertension, n (%)	5 (10.4)	6 (12.5)	>0.999	0.065
Diabetes mellitus, n (%)	3 (6.2)	3 (6.2)	>0.999	0.000

Continuous variables are presented as mean ± SD; categorical variables as n (%). SMD indicates standardized mean difference.

### Perioperative outcomes

Perioperative outcomes in the matched cohort are summarized in **Table 2**. Compared with SR, MWA was associated with significantly shorter operative duration (median 18.0 minutes [IQR, 16.0-21.0] vs. 63.5 minutes [55.0-75.0]; P <0.001), lower intraoperative blood loss (1.5 mL [1.0-2.0] vs. 53.5 mL [37.8-64.2]; P <0.001), and shorter hospital stay (1.0 day [1.0-2.0] vs. 5.0 days [4.0-6.0]; P <0.001).

**Table 2:** Perioperative outcomes, clinical efficacy, and complications in the matched cohort.

Variable	MWA (n=48)	SR (n=48)	P value
Operation duration (min)	18.0 (16.0-21.0)	63.5 (55.0-75.0)	<0.001
Intraoperative blood loss (mL)	1.5 (1.0-2.0)	53.5 (37.8-64.2)	<0.001
Hospital stay (days)	1.0 (1.0-2.0)	5.0 (4.0-6.0)	<0.001
VRR at 3 months (%)	60.7 (58.4-64.9)	NA	NA
VRR at 6 months (%)	75.3 (71.3-80.5)	NA	NA
VRR at 12 months (%)	86.2 (79.1-89.1)	NA	NA
Symptom score, pre-treatment	5.0 (4.0-6.0)	5.0 (3.0-6.2)	0.678
Symptom score, 12 months	1.0 (1.0-2.0)	1.0 (0.0-2.0)	0.221
Residual/recurrence at 12 months, n (%)	3 (6.2)	1 (2.1)	0.617
Overall thyroid hormone dependence, n (%)	1 (2.1)	25 (52.1)	<0.001
Planned replacement after total thyroidectomy	0	14/14 (100)	NA
New-onset hypothyroidism after organ-preserving procedures	1/48 (2.1)	11/34 (32.4)	<0.001
Hoarseness, n (%)	2 (4.2)	1 (2.1)	>0.999
Hypocalcemia, n (%)	0 (0.0)	3 (6.2)	0.242

Continuous variables are presented as median (IQR); categorical variables as n (%). NA, not applicable; VRR, volume reduction rate. VRR was not applicable to SR because the target nodule was surgically removed.

### Clinical efficacy

In the MWA group, median VRR increased from 60.7% at 3 months to 75.3% at 6 months and 86.2% at 12 months. This exceeded the commonly used 50% threshold for technical and clinical success after thyroid thermal ablation (14–16). VRR was not calculated for SR because the target nodule was anatomically removed. Patient-reported symptom scores were comparable between groups before treatment (median 5.0 vs. 5.0; P = 0.678) and decreased substantially by 12 months in both the MWA group (median 5.0 to 1.0; P < 0.001) and the SR group (median 5.0 to 1.0; P < 0.001). The degree of symptom improvement did not differ between groups (P = 0.782), and 12-month symptom scores remained low and similar in both groups (median 1.0 vs. 1.0; P = 0.221). Residual or recurrent disease was detected in 3 MWA patients (6.2%) and 1 SR patient (2.1%; P = 0.617).

### Thyroid hormone dependence and complications

Overall thyroid hormone dependence at 12 months occurred in 1 MWA patient (2.1%) and 25 SR patients (52.1%; P <0.001). In the MWA group, the single case represented new-onset subclinical hypothyroidism requiring low-dose levothyroxine. In the SR group, overall hormone dependence comprised planned replacement after total thyroidectomy (14/14, 100%) and new-onset hypothyroidism after organ-preserving procedures (11/34, 32.4%). Among organ-preserving surgical procedures, new-onset hypothyroidism occurred in 11 of 25 lobectomy patients and 0 of 9 subtotal thyroidectomy patients.

In the pre-specified sensitivity analysis excluding total thyroidectomy patients, new-onset hypothyroidism remained significantly less frequent after MWA than after organ-preserving surgery (1/48 [2.1%] vs. 11/34 [32.4%]; P <0.001). In a secondary analysis that separately re-matched MWA patients one-to-one to organ-preserving surgical patients (31 matched pairs), new-onset hypothyroidism remained significantly less frequent after MWA (1/31 [3.2%] vs. 9/31 [29.0%]; P = 0.012), although covariate balance was slightly reduced (maximum standardized mean difference 0.34, relative to the organ-preserving surgical index group) given the smaller organ-preserving pool. Hoarseness occurred in two MWA patients (4.2%) and one SR patient (2.1%; P > 0.999). Hypocalcemia occurred only after SR (3/48, 6.2%), although the between-group difference was not statistically significant. No hematoma, wound infection, or perioperative death occurred in either group.

## Discussion

### Main findings

In this propensity score-matched retrospective cohort study, MWA was associated with lower perioperative burden and less postoperative thyroid hormone dependence than SR among selected patients with symptomatic benign thyroid nodules. At 12 months, the MWA group showed substantial nodule shrinkage, low residual symptom burden, and a low rate of hormone dependence. Importantly, the SR group’s overall hormone-dependence rate was a composite endpoint that included both planned replacement after total thyroidectomy and new-onset hypothyroidism after organ-preserving surgery. Therefore, the overall comparison should be interpreted as a pragmatic comparison of treatment strategies rather than a pure biological contrast between MWA and a uniform surgical procedure.

The sensitivity analysis excluding total thyroidectomy was central to interpretation. Even after removing patients in whom hormone replacement was inevitable, new-onset hypothyroidism remained less frequent after MWA than after lobectomy or subtotal thyroidectomy (5, 17). This finding supports the functional preservation advantage of MWA, while also acknowledging that residual confounding by disease extent, multinodularity, and surgical selection cannot be fully eliminated in a retrospective study. Importantly, the secondary analysis that re-matched MWA patients one-to-one to organ-preserving surgical patients achieved less optimal covariate balance than the primary matched cohort, with a maximum standardized mean difference of 0.34—exceeding our pre-specified threshold of 0.20 for adequate balance—because the organ-preserving surgical pool available for matching was small (34 patients), with the residual imbalance concentrated in the baseline TSH and comorbidity covariates. The functional preservation findings from this re-matched analysis should therefore be interpreted with appropriate caution, as the residual imbalance could have biased the comparison in either direction; this analysis is best regarded as supportive rather than confirmatory. Reassuringly, the direction of the functional preservation advantage was consistent across the primary matched cohort, the total-thyroidectomy-excluded sensitivity analysis, and the re-matched cohort, but its magnitude requires confirmation in larger prospective cohorts.

### Comparison with previous evidence

Our 12-month median VRR of 86.2% after MWA is at the upper end of, and somewhat above, pooled estimates for MWA, in which the 12-month VRR has generally been reported in the range of approximately 77% to 80% (for example, 76.9% and 80.0% in two head-to-head meta-analyses and 76.1% in a multicenter cohort of large nodules) (18, 21, 23). Volume reduction after MWA continues over time, with rapid early shrinkage followed by a slower phase (20), and a single-center cohort with more than 48 months of follow-up reported a VRR of approximately 97% (22), suggesting that our 12-month value likely underestimates the eventual response. In head-to-head comparisons, RFA and MWA show broadly comparable efficacy and safety, with some meta-analyses reporting a modestly higher 12-month VRR for RFA (standardized mean difference 0.38) but no meaningful difference in symptom improvement (symptom-score standardized mean difference 1.46 vs. 1.45 for RFA vs. MWA in one analysis) (19, 21). This symptomatic benefit is also reflected in routine clinical practice, in which most patients report symptom resolution after percutaneous thermal ablation (24). Against this background, the present study adds value not by reaffirming the efficacy of MWA, which is already well established, but by focusing on postoperative thyroid function and by separating planned replacement after total thyroidectomy from new-onset hypothyroidism after procedures that preserve part or all of the thyroid gland (25).

Our findings are also consistent with contemporary guideline recommendations that support thermal ablation for selected symptomatic, cytologically benign nodules (11–13). In clinical practice, the decision between MWA and surgery is influenced by nodule size, location, multinodularity, cosmetic concerns, patient preference, institutional expertise, and the need for definitive histology (26). Therefore, MWA should not be interpreted as replacing surgery for all benign thyroid nodules; rather, it may be particularly suitable for patients with small-to-moderate nodules who value organ preservation and wish to avoid general anesthesia, cervical incision, or hospitalization (27).

### Clinical interpretation

A key issue raised by this study is the definition of postoperative thyroid hormone dependence. Planned levothyroxine after total thyroidectomy is an expected consequence of complete gland removal, whereas new-onset hypothyroidism after lobectomy, subtotal thyroidectomy, or MWA represents a postoperative functional outcome. Combining these outcomes can overstate the apparent difference between MWA and surgery if not clearly stratified. For this reason, we reported both the overall pragmatic endpoint and the organ-preserving sensitivity analysis. This approach better reflects how clinicians and patients discuss treatment options: surgery may be anatomically definitive, but its functional consequences depend strongly on the extent of resection.

The relatively small baseline nodule volume in this cohort should also guide interpretation. The average preoperative volume was approximately 3 mL, indicating that the findings are most applicable to small-to-moderate symptomatic or cosmetically concerning nodules rather than very large multinodular goiters. Some nodules of this size can cause clinically relevant symptoms when located near the trachea, esophagus, recurrent laryngeal nerve region, or anterior neck. Both treatments produced substantial and comparable symptom relief from a similar baseline symptom burden, supporting the clinical relevance of treatment in this selected population.

### Limitations and strengths

This study has limitations. First, it was retrospective and single-center, and PSM can address only measured covariates. Second, the sample size was modest, limiting precision for uncommon complications and subgroup analyses. Third, surgical extent was heterogeneous and was influenced by multinodularity and contralateral nodular burden. Although we stratified thyroid hormone outcomes by resection type and performed sensitivity analyses excluding total thyroidectomy, residual treatment-selection bias remains possible. This limitation was most pronounced in the secondary re-matched organ-preserving analysis, in which the small surgical pool constrained the achievable covariate balance (maximum standardized mean difference 0.34); accordingly, that analysis should be regarded as supportive rather than confirmatory. Fourth, symptom burden was captured with a simple institutional 10-point patient-reported scale rather than a formally validated thyroid-specific symptom or quality-of-life instrument, which may limit comparability with other studies. Fifth, known autoimmune thyroiditis and abnormal preoperative thyroid function were excluded, but antibody status was not uniformly available for all patients; occult thyroid autoimmunity may have contributed to postoperative hypothyroidism in some cases. Sixth, contrast-enhanced ultrasound and serial de-identified representative images were not routinely archived in a standardized manner during the retrospective study period, so imaging response was evaluated through measured ultrasound volumes rather than illustrative image panels. Finally, follow-up was limited to 12 months, and longer observation is needed to assess durability, regrowth, and delayed thyroid dysfunction.

The study also has strengths. Consecutive patients were identified from an institutional registry, all matched patients completed 12-month follow-up, and matching achieved acceptable balance across the pre-specified baseline covariates. The primary functional outcome was reported in a clinically transparent manner, distinguishing planned post-thyroidectomy hormone replacement from new-onset hypothyroidism. This stratified reporting directly addresses a common limitation in comparative studies of thyroid ablation and surgery.

## Conclusion

In selected patients with small-to-moderate symptomatic benign thyroid nodules, ultrasound-guided MWA achieved substantial 12-month volume reduction with low residual symptom burden, shorter perioperative recovery, and lower postoperative thyroid hormone dependence than surgical resection. The functional advantage persisted when planned hormone replacement after total thyroidectomy was excluded, although residual confounding and the retrospective single-center design limit causal inference. Prospective multicenter studies with longer follow-up, standardized symptom and quality-of-life measures, autoimmune thyroiditis assessment, and formal cost-effectiveness analyses are needed to define the optimal role of MWA in benign thyroid nodule management.

## Data Availability

The raw data supporting the conclusions of this article will be made available by the authors, without undue reservation.
